# Computed Tomography Investigation of First Metacarpal Subsidence After Suture Button Suspensionplasty: A Report of Two Cases

**DOI:** 10.7759/cureus.71499

**Published:** 2024-10-14

**Authors:** Shunpei Hama, Masataka Yasuda

**Affiliations:** 1 Department of Orthopedic Surgery, Yodogawa Christian Hospital, Osaka, JPN; 2 Department of Orthopedic Surgery, Baba Memorial Hospital, Sakai, JPN

**Keywords:** 1st carpometacarpal arthritis, computed tomography (ct ), mini tight rope, suture button suspensionplasty, thumb shortening

## Abstract

First metacarpal subsidence often occurs after suture button suspensionplasty (SBS) for first carpometacarpal joint arthritis. In this study, we describe computed tomography (CT) findings in two cases of first metacarpal subsidence after SBS. Case 1 was a 51-year-old right-handed male. At the final follow-up two years post-SBS, no noticeable thumb shortening was observed. CT imaging at the last follow-up showed a slight expansion of the hole in the first and second metacarpals where the suture had passed. Case 2 was a 58-year-old right-handed male. Thumb shortening was noticed three months and three weeks after SBS. At the final follow-up, two years and seven months post-surgery, CT imaging revealed significant expansion of the hole in a conical shape in the second metacarpal where the suture had passed. Our findings suggest that the expansion of the foramen where the suture passed through the metacarpals may contribute to the first metacarpal subsidence after the surgery.

## Introduction

First carpometacarpal (CMC) joint osteoarthritis (OA) is a main condition that may extremely impair daily life [1]. The first CMC joint is composed of two saddle-shaped bones that articulate perpendicularly to each other [1]. The composition of the joint allows a wide range of motion for pinching and gripping [1]. Due to the unique anatomy of the first CMC joint, the joint is vulnerable to instability [1] and descends into OA [2]. When nonoperative treatments fail, the surgical intervention can be considered [1]. There are a variety of surgeries for first CMC joint OA, including CMC joint arthrodesis [3], trapeziectomy with ligament reconstruction and tendon interposition (LRTI) [4], and first metacarpal extension osteotomy [5]. Suture button suspensionplasty (SBS) is also one of the options for surgical intervention. SBS using the Mini-Tight-Rope (Arthrex, Naples, FL) in combination with trapeziectomy has been reported as a minimally invasive technique for treating OA at the first CMC joint [1]. SBS accomplishes stability like K-wire fixation after trapeziectomy [6] and the patients can begin rehabilitation faster than the K-wire fixation. However, first metacarpal subsidence is a common issue after this surgery, and the reasons for this phenomenon remain unclear. To the best of our knowledge, there have been no reports about the cause of the subsidence of the first metacarpal and no reports where surgeons used computed tomography (CT) to analyze the cause of the first metacarpal subsidence following SBS. If we find the cause of this subsidence using CT, surgeons give the patient the cause of the subsidence more precisely. In this report, we present two cases of metacarpal subsidence after SBS, and CT imaging revealed cavitation in the metacarpals where the suture passed through. The patients provided informed consent for the publication of this case report and any accompanying images.

## Case presentation

Case 1

**Figure 1:**
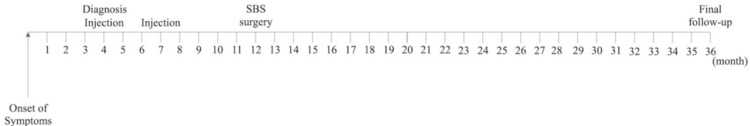
Timeline including key clinical events of case 1.

**Figure 2:**
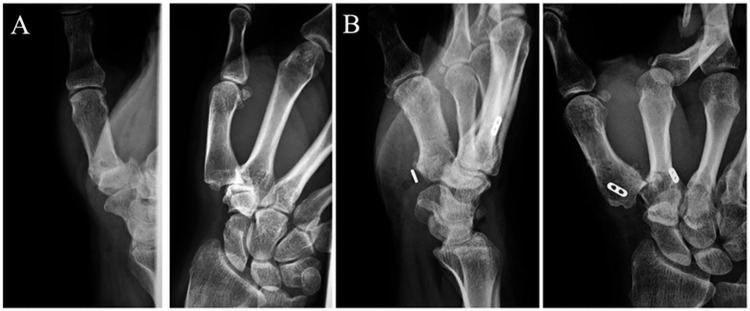
(A) Posteroanterior and lateral radiographs of the preoperative right first carpometacarpal (CMC) joint. (B) Posteroanterior and lateral radiographs of the right first CMC joint just after the surgery.

**Figure 3:**
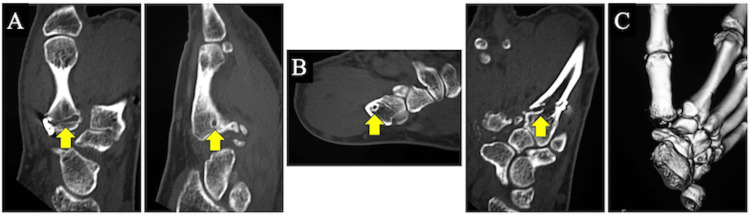
(A) Coronal and sagittal computed tomography (CT) images of the right first metacarpal two years after the surgery. (B) Horizontal and sagittal CT images of the right second metacarpal two years after the surgery (arrows: the foramen in the metacarpals where the rope passed). (C) Three-dimensional CT image of the right hand two years after the surgery.

A 51-year-old right-handed male was referred to our hospital due to right first CMC joint pain persisting for four months (Figure 1). He worked in plastic molding. His pinch strength on the right side was 3.5 kg, which was weaker than the left side (7.0 kg). Physical examination revealed tenderness in the right first CMC joint, and the grinding test was slightly positive. The Disabilities of the Arm, Shoulder, and Hand (DASH) score was 13.8. Radiographs of the right first CMC joint showed Eaton stage 3 OA (Figure 2A). Initially, we injected triamcinolone acetonide into the first CMC joint and provided a commercial brace. However, despite repeating the injection twice at a three-month interval due to recurrent pain, conservative therapy did not fully alleviate the thumb pain over eight months. Consequently, we planned an SBS using the Mini-Tight-Rope. During surgery, we resected the trapezium and passed the suture from the insertion area of the abductor pollicis longus in the first metacarpal to the base of the second metacarpal (Figure 2B). Postoperative immobilization was not performed, and the patient, who had a physically demanding job, required pain relievers. At the final follow-up two years post-surgery, both right and left pulp pinch strengths were 7.0 kg, and the DASH score was 12. No noticeable thumb shortening was observed. The metacarpal subsidence ratio, defined as the percentage of postoperative trapezial space height compared to preoperative trapezial height [1], was 58.7% immediately after surgery and 50.7% at the final follow-up. CT imaging at the last follow-up showed a slight expansion of the hole in the first and second metacarpals where the suture had passed (Figures 3A-3C).

Case 2

**Figure 4:**
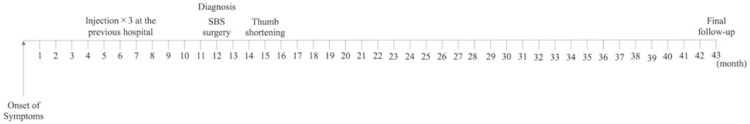
Timeline including key clinical events of case 2.

**Figure 5:**
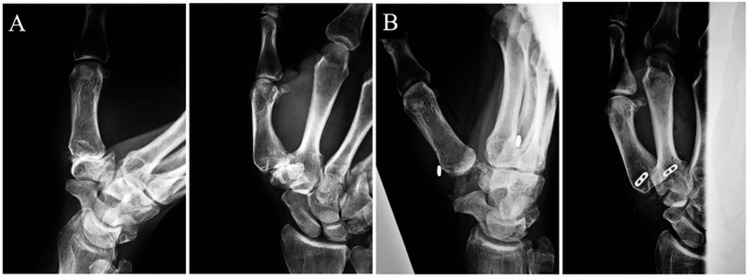
(A) Posteroanterior and lateral radiographs of the right first carpometacarpal (CMC) joint before the surgery. (B) Posteroanterior and lateral radiographs of the postoperative right first CMC joint.

**Figure 6:**
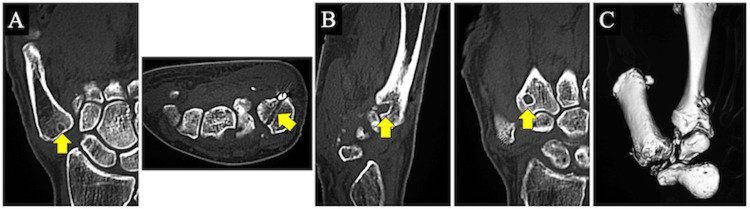
(A) Sagittal and horizontal computed tomography (CT) images of the right first metacarpal two years and seven months after the surgery. (B) Sagittal and coronal image CT images of the right second metacarpal two years and seven months after the surgery. The CT images showed the huge expanding of the hole in a conical shape in the second metacarpal (arrows: the foramen in the metacarpals where the rope passed). (C) Three-dimensional CT image of the right first carpometacarpal joint two years at the last follow up.

A 58-year-old right-handed male was referred to our hospital due to right first CMC joint pain persisting for one year (Figure 4). Triamcinolone acetonide had been injected three times before his first visit to our hospital. The patient had multiple Heberden's nodes. Right and left pulp pinch strengths were 7.4 kg and 6.3 kg, respectively. The grinding test was slightly positive, and tenderness in the right first CMC joint was mild. Radiographs showed Eaton stage 3 OA of the right first CMC joint (Figure 5A). The DASH score was 10.3. The same surgical procedure as in Case 1 was performed (Figure 5B), and pregabalin was prescribed postoperatively. Although the patient returned to work approximately three weeks after surgery, thumb shortening was visually noticed three months later. At the final follow-up, two years and seven months post-surgery, pulp pinch strengths were 5.6 kg on the right side and 6.6 kg on the left side, and the final DASH score was 8.6. Metacarpal subsidence ratios were 104.0% immediately after surgery (Figure 5B) and 50.7% at the final follow-up. CT imaging at the last follow-up revealed significant expansion of the hole in a conical shape in the second metacarpal where the suture had passed (Figures 6A, 6B). No contact between the first metacarpal and the scaphoid was observed (Figure 6C).

## Discussion

The CT scans of the two current cases following SBS surgery revealed a cavitation formed by the ropes within the metacarpals. In case 1, the expansion of this hole was minimal, thus preventing significant shortening of the thumb. However, in case 2, the broader opening created by the rope compared to case 1 may have contributed to thumb shortening. This report sheds light on what occurs in cases of first metacarpal subsidence after SBS surgery, adding to the limited references that investigate the causes of metacarpal subsidence using CT.

SBS surgery is a version of the hematoma distraction arthroplasty in which instead of using a K-wire to suspend the first metacarpal [7]. Mini-Tight-Rope utilizes a 2.0 fiber wire and has been biomechanically shown to provide greater resistance to metacarpal subsidence under immediate loading compared to LRTI in cadaveric models [2]. This device allows immediate thumb range of motion and, complications associated with K-wire including pin site infection and migration can be prevented in SBS surgery. In addition, tendon weaves or transfers, which take time to heal and establish stability, are unnecessary in the surgery [7]. After LRTI procedures, first metacarpal subsidence often occurs, with an incidence rate of 21% after an average follow-up of 32 months. Nevertheless, this subsidence is believed to have little impact on clinical outcomes [8]. However, metacarpal subsidence after trapeziectomy has long been a concern. Due to the anxiety about the subsidence hand surgeons have either continued suspending the first metacarpal, inserted tissue into the trapeziectomy space, or performed a combination of both [7].

When the metacarpal subsidence ratio is defined as the percentage of postoperative trapezial space height relative to preoperative trapezial height, the subsidence ratio of the trapezium was reported to be 25% after a minimum two-year follow-up and 29% [1] in a study with a mean five-year follow-up after SBS [7]. The subsidence rates at the final follow-up in the present report were higher than those reported by the previous report. The cause of the subsidence could be the expansion of the hole where the rope passed. If the hole is enlarged, the metacarpal fracture could occur. Yao et al. reported a second metacarpal fracture in the two-year follow-up after SBS surgery [1]. If the hole is enlarged, the metacarpal fracture could occur. Yao et al. reported a second metacarpal fracture in the two-year follow-up after SBS surgery [1]. Khalid et al. also reported a second metacarpal fracture after SBS surgery. They noted that the hole that had been drilled for the rope was larger in diameter on the revision surgery than it was originally drilled. The reason for the enlargement of the hole was thought to be a rotation of the rope within the bone and/or osteolysis [9]. A case of a 79-year-old female with sudden complete subsidence of the first metacarpal six months after SBS, attributed to the breakage of the Mini-Tight-Rope, has also been documented [10]. The cases presented here involved male individuals in physically demanding occupations, and this occupational factor might have contributed to trapezial space collapse.

## Conclusions

One limitation of this case report is the possibility that the Mini-Tight-Rope may have broken along with the formation of the cavitation in the metacarpal. Based on the findings of the expanded foramen, we speculate that there was considerable movement of the rope before any potential breakage occurred. This case report offers insight into the phenomenon of first metacarpal subsidence following SBS surgery and identifies the cavitation in the metacarpals where the rope passes as one of the contributing factors to this subsidence.

## References

[1] Yao J, Song Y (2013). Suture-button suspensionplasty for thumb carpometacarpal arthritis: a minimum 2-year follow-up. J Hand Surg Am.

[2] Desai MJ, Brogan DM, Richard MJ, Mithani SK, Leversedge FJ, Ruch DS (2016). Biomechanical comparison of suture-button suspensionplasty and LRTI for basilar thumb arthritis. Hand (N Y).

[3] Vermeulen GM, Brink SM, Slijper H, Feitz R, Moojen TM, Hovius SE, Selles RW (2014). Trapeziometacarpal arthrodesis or trapeziectomy with ligament reconstruction in primary trapeziometacarpal osteoarthritis: a randomized controlled trial. J Bone Joint Surg Am.

[4] Vinycomb T, Crock J (2013). Trapeziectomy with ligament reconstruction and tendon interposition: a minimum 10-year follow-up. Hand Surg.

[5] Parker WL, Linscheid RL, Amadio PC (2008). Long-term outcomes of first metacarpal extension osteotomy in the treatment of carpal-metacarpal osteoarthritis. J Hand Surg Am.

[6] Yao J, Zlotolow DA, Murdock R, Christian M (2010). Suture button compared with K-wire fixation for maintenance of posttrapeziectomy space height in a cadaver model of lateral pinch. J Hand Surg Am.

[7] Yao J, Cheah AE (2017). Mean 5-year follow-up for suture button suspensionplasty in the treatment of thumb carpometacarpal joint osteoarthritis. J Hand Surg Am.

[8] Yang SS, Weiland AJ (1998). First metacarpal subsidence during pinch after ligament reconstruction and tendon interposition basal joint arthroplasty of the thumb. J Hand Surg Am.

[9] Khalid M, Jones ML (2012). Index metacarpal fracture after tightrope suspension following trapeziectomy: case report. J Hand Surg Am.

[10] Sonoda LA, Jones NF (2017). Failed suture button suspensionplasty of the thumb carpometacarpal joint salvaged using pyrocarbon arthroplasty. J Hand Surg Am.

